# Dealing with feelings in adolescence: Cognitive reappraisals in unpleasant and pleasant emotional events and their associations with subjective well‐being

**DOI:** 10.1111/jora.70162

**Published:** 2026-02-25

**Authors:** F. Sternke, S. Nestler, E. S. Blanke, U. Kunzmann

**Affiliations:** ^1^ Wilhelm Wundt Institute for Psychology, Lifespan Psychology Lab University of Leipzig Leipzig Germany; ^2^ Institute for Psychology, Statistics and Psychological Methods Working Unit University of Münster Münster Germany

**Keywords:** adolescence, daily diary, development, reappraisal, subjective well‐being

## Abstract

Adolescence is a period of heightened exposure to both unpleasant and pleasant events, requiring effective emotion regulation. Cognitive reappraisal is particularly beneficial, yet research has typically examined its role either in unpleasant or in pleasant situations, rarely considering both simultaneously within individuals. In this 28‐day daily diary study, we investigated whether cognitive reappraisal in unpleasant and pleasant events each uniquely contributes to subjective well‐being, and, given cognitive maturation during adolescence, whether these associations become stronger with increasing age. A sample of 122 adolescents (15–19 years; *M* = 17.01, *SD* = 1.42) reported their end‐of‐day subjective well‐being and the use of eight cognitive reappraisal strategies for the day's most unpleasant and most pleasant events. On a within‐person level, both types of reappraisal predicted higher subjective well‐being, even when simultaneously included in the model. Unexpectedly, these effects did not vary by age. On a between‐person level, reappraisal in unpleasant and pleasant events was each associated with higher subjective well‐being, but not when analyzed jointly, due to shared variance between both types of reappraisal. The within‐person findings highlight that regulating emotions in both unpleasant and pleasant events uniquely contributes to adolescent well‐being, emphasizing the importance of context on emotion regulation in adolescents.

## INTRODUCTION

Adolescence, typically defined as ages 10 to 19 (World Health Organization, 2019), is marked by substantial biological, psychological, and social changes (Gilbert, 2012). These developmental changes are accompanied by frequent and often intensified emotional experiences, both unpleasant and pleasant^1^ (Larson et al., 1980), thereby increasing adolescents' need to regulate their emotions effectively (Gross, 2015; Tamir, 2016).

On a general level, emotion regulation refers to “the processes by which individuals influence which emotions they have, when they have them, and how they experience and express these emotions” (Gross, 1998, p. 275). Among the wide range of emotion regulation strategies, cognitive reappraisal has been considered as particularly effective (Boemo et al., 2022; Webb et al., 2012). Cognitive reappraisal involves changing one's perception of the meaning or self‐relevance of a situation to alter its emotional impact (Gross, 2015). It is employed in response to both unpleasant and pleasant events and may involve reappraising an event as less negative than it initially appears (McRae, Gross, et al., 2012) or actively focusing on the positive meaning and consequences of a pleasant situation (Feldman et al., 2008).

Across both unpleasant and pleasant events, cognitive reappraisal has been theoretically and empirically linked to higher subjective well‐being, defined by high positive affect, low negative affect, and high life satisfaction (Diener, 1984; Heiy & Cheavens, 2014). Given that the two types of cognitive reappraisal have typically been investigated in isolation, however, it remains unclear whether they uniquely contribute to adolescents' subjective well‐being when considered simultaneously. For example, it is feasible that regulating emotions in unpleasant situations is sufficient for ensuring subjective well‐being, while emotion regulation in pleasant situations adds no additional benefit. However, it is also possible that regulating emotions in pleasant situations makes a distinct and independent contribution to subjective well‐being. This is of particular importance as daily life is characterized by not only unpleasant or only pleasant events, but by both, and hence by cognitive reappraisal in unpleasant and in pleasant events.

Therefore, in this daily diary study, we sought to extend prior work by jointly examining both types of cognitive reappraisal and isolating their unique contributions to adolescents' daily subjective well‐being. Considering the rapid cognitive maturation in adolescence (Keshavan et al., 2014), we further examined whether the within‐person associations between daily cognitive reappraisal and subjective well‐being strengthen with increasing age, indicating greater effectiveness as adolescents grow older. To this end, we extend prior laboratory‐based research (e.g., McRae, Gross, et al., 2012; Silvers et al., 2012, 2015) by a diary study in the context of which cognitive reappraisal use was assessed in relation to naturally occurring unpleasant and pleasant events in everyday life.

### Cognitive reappraisal use and subjective well‐being in adolescence

Emotions are a key evolutionary mechanism that enables humans to adapt to environmental changes, thereby possessing an innate adaptive value (Smith & Lazarus, 1990). At the same time, emotions often require regulation in order to achieve personal goals and maintain subjective well‐being, both one's own or that of others (Gross, 2015; Tamir, 2016). According to Diener's (1984) definition, subjective well‐being encompasses an affective component of low negative affect and high positive affect, and a cognitive‐evaluative component of high life satisfaction. These components are closely related and tend to form a single factor, pointing to a coherent construct (e.g., Ronen et al., 2016; Schunk et al., 2022).

Adolescents undergo marked changes in their experience of subjective well‐being: They experience stronger negative and positive affect and greater affective variability compared to children and adults (Larson et al., 1980; Reitsema et al., 2022). An explanation could be that adolescents' brain regions for affective reactivity, such as the amygdala and the nucleus accumbens, develop earlier than those for regulation (Gilbert, 2012). Additionally, adolescents face new types of unpleasant events, such as increasing school stress (Byrne et al., 2007) or uncertainty of one's identity (Kroger et al., 2009). However, adolescence is also characterized by several new types of pleasant events, such as profound friendships (Rice & Mulkeen, 1995) and the first romantic relationships (Zimmer‐Gembeck, 1999). Experiencing such pleasant emotions or affect can prevent the occurrence of negative affect (Moneta et al., 2012) and stress (Ong et al., 2006), and even help adolescents strengthen their friendships (Šutić et al., 2025).

In unpleasant events, an adolescent might, for example, downregulate their anxiety in front of peers to keep their reputation, while in pleasant events, an adolescent might share good news with friends to increase their own and their friends' joy. Hence, effectively regulating one's emotions in both unpleasant and pleasant events is just as much a part of everyday experience as emotions themselves (Heiy & Cheavens, 2014) and of greatest importance.

In our study, we focus on cognitive reappraisal, as it is regarded as particularly effective for both the upregulation of pleasant emotions and the downregulation of unpleasant emotions (Boemo et al., 2022; Webb et al., 2012). Both in unpleasant and pleasant events, cognitive reappraisal comprises several subtypes. In unpleasant events, these can be relativizing, positive (i.e., seeing positive outcomes or the situation as less negative), or detached reappraisal (i.e., self‐distancing from the situation) (McRae, Ciesielski, & Gross, 2012; Webb et al., 2012). In pleasant events, reappraisal strategies either make events appear more pleasant (e.g., “I thought that the situation was a very special moment”) or of greater relevance to oneself (e.g., “I thought about how my strengths contributed to the situation”). This is to reflect that reappraisal aims at changing one's emotions by cognitively altering the meaning or self‐relevance of the situation (Gross, 2015). Furthermore, as reappraisal strategies in pleasant events fall under the umbrella term of savoring, which has been linked to improved well‐being (Bryant & Veroff, 2007; Jose et al., 2012; Quoidbach et al., 2010), our items of cognitive reappraisal excluded any upregulation of unpleasant emotions (e.g., catastrophizing) or any downregulation of pleasant emotions (e.g., dampening). In some questionnaires (e.g., Feldman et al., 2008) and studies (e.g., Chadwick et al., 2020), cognitive reappraisal in pleasant events is referred to as positive rumination—in others, it is referred to as reappraisal (Mueller et al., 2023). We decided to use the term “reappraisal”, as it focuses on the content of thoughts, whereas rumination denotes strategies for deploying attention in a repetitive and affect‐focused manner (Gross, 1998).

### Past empirical evidence for cognitive reappraisal in unpleasant events

Because unpleasant events induce unpleasant emotions (Moberly & Watkins, 2008), which are linked to fewer pleasant emotions (Brose et al., 2015), cognitive reappraisal in unpleasant events is likely most often used to regulate unpleasant emotions. In adults, such reappraisal is associated with higher subjective well‐being, including higher positive affect and lower negative affect in both trait‐ and state‐level studies, considering both the between‐ and within‐person level (e.g., Heiy & Cheavens, 2014; Newman & Nezlek, 2022; Schunk et al., 2022; Webb et al., 2012). In trait studies, general tendencies across longer time frames are assessed (e.g., across months or years), often investigating between‐person effects, that is, elucidating whether people who have the tendency to use reappraisal are also people who experience higher subjective well‐being. In state studies, reappraisal use and well‐being are assessed during a short time frame (e.g., hours or one day), usually repeatedly, investigating within‐person whether moments of reappraisal use are also moments of higher well‐being. Results for between‐person and within‐person investigations can thus diverge (e.g., Boemo et al., 2022; Verzeletti et al., 2016), as seems to be the case in adolescent emotion regulation.

Specifically, in adolescents, similar patterns as in adults appear at the between‐person level in trait studies: greater use of reappraisal in unpleasant situations is linked to higher positive affect, lower negative affect, and greater life satisfaction (Verzeletti et al., 2016; Wante et al., 2017; Zou et al., 2022). In contrast, the one existing study at the state level in adolescents with within‐person analyses (Lennarz et al., 2018) has largely failed to detect such associations. Lennarz et al. (2018) suggested that the absence of a relationship may reflect low levels and, thus, limited variability of negative affect, as adolescents in their study only provided weekend reports. It thus remains unclear whether the link between reappraisal in unpleasant situations and subjective well‐being in adolescents holds for within‐person associations at the state level.

### Past empirical evidence for cognitive reappraisal in pleasant events

As pleasant events entail more pleasant emotions, which help to further reduce unpleasant emotions (Moneta et al., 2012), cognitive reappraisal in pleasant events is typically used to regulate pleasant emotions. In adults, in both state‐ and trait‐level studies, such reappraisal is associated with higher life satisfaction (Quoidbach et al., 2010), higher positive affect (Li et al., 2017; Newman & Nezlek, 2022), and lower negative affect (Hurley & Kwon, 2012; Newman & Nezlek, 2022).

In adolescents, on a trait‐level, reappraisal in pleasant events has been linked to higher life satisfaction (Gomez‐Baya et al., 2018) and both higher positive affect and life satisfaction (Chadwick et al., 2020). On a state‐level, reappraisal in pleasant events (jointly with other strategies focused on upregulation) has been associated with higher positive affect (Gentzler et al., 2013). However, both on a trait‐ and on a state level, these associations have typically been examined independently of reappraisal in unpleasant contexts. The only study to date that has investigated cognitive reappraisal in both unpleasant and pleasant events assessed reappraisal in pleasant events in a somewhat unusual manner, suggesting that it likely also involved a downregulation of pleasant emotions (Mueller et al., 2023). But as cognitive reappraisal is generally considered an adaptive strategy (Heiy & Cheavens, 2014), including the dampening of pleasant emotions makes the interpretation of its links with subjective well‐being difficult.

Thus, it remains largely unclear whether the use of cognitive reappraisal in pleasant situations in the sense of savoring offers additional benefits beyond the use of cognitive reappraisal in unpleasant situations with the purpose of down‐regulating negative affect. Given that pleasant emotions serve important adaptive functions (Fredrickson, 1998), occur frequently, and are regulated just as often as unpleasant emotions (Heiy & Cheavens, 2014), we expect that the regulation of pleasant emotions, such as through cognitive reappraisal, should make a distinct and independent contribution to adolescents' subjective well‐being.

### The importance of examining links of cognitive reappraisal on a within‐person level

Investigating within‐person associations is particularly important when studying emotion regulation, as regulatory processes unfold in response to situational demands and fluctuate across time. In adolescence, evidence for associations between cognitive reappraisal and subjective well‐being at the within‐person level is sparse, despite robust trait‐level findings. This discrepancy suggests that between‐person associations may not adequately capture adolescents' momentary use of cognitive reappraisal in daily life. One potential reason might be that reflecting on one's own trait‐level of cognitive reappraisal may be influenced by one's self‐concept (e.g., “Am I a reappraiser?”). Furthermore, only by employing repeated state‐level measures can potentially meaningful contextual variations, such as the amount of unpleasant and pleasant daily events, be included (Li et al., 2017; Newman & Nezlek, 2022). Only when a high amount of unpleasant events or a low amount of pleasant events have occurred that day, might cognitive reappraisal in pleasant events be linked to lower negative affect or higher positive affect (Li et al., 2017; Newman & Nezlek, 2022).

In designs such as daily diaries, both state‐ and trait components (as aggregated states) can be captured, and therefore, daily diaries are being employed more often. Such studies indicate, for example, that cognitive reappraisal in unpleasant events is associated with less substance use (Weiss et al., 2017) and less fear of missing out (Hartanto et al., 2022), while cognitive reappraisal in pleasant events is linked to lower levels of depression in young adults (Li et al., 2017). At the same time, examining between‐person associations remains informative, as individuals differ in their general tendency to use cognitive reappraisal. Therefore, the present study considers both within‐ and between‐person associations, with a primary focus on within‐person links to capture the dynamic role of cognitive reappraisal in adolescents' everyday lives.

### Age differences in cognitive reappraisal effectiveness during adolescence

During adolescence, marked psychosocial and biological changes occur. Family‐related factors such as family climate shape the development of cognitive reappraisal (Ogbaselase et al., 2022)⸺however, rapidly advancing neurocognitive maturation likely also plays a crucial role (Gilbert, 2012; Keshavan et al., 2014). During this period, the adolescent brain undergoes extensive structural and functional reorganization, particularly in prefrontal and frontoparietal networks during late adolescence (Keshavan et al., 2014). These prefrontal maturations lead to a significant improvement in executive functions (Keshavan et al., 2014), which are a set of general‐purpose regulatory processes, such as updating information in working memory (Toh et al., 2024). Better executive functioning, in turn, likely enhances adolescents' capacity to reflect on and reinterpret emotional events, thereby facilitating more effective use of cognitive reappraisal, consistent with both neuroimaging evidence (Messina et al., 2015) and behavioral findings showing age‐related increases in reappraisal use (Toh et al., 2024). Thus, cognitive reappraisal is expected to become increasingly effective as adolescents grow older.

Studies investigating age differences in cognitive reappraisal in adolescents have primarily relied on laboratory‐based psychological experiments. These studies have typically instructed participants to use cognitive reappraisal strategies to downregulate negative affect in response to emotional stimuli and compared this condition to control conditions, either without emotion regulation instructions (e.g., Ahmed et al., 2018; McRae, Gross, et al., 2012; Nook et al., 2020; Silvers et al., 2012, study 1) or with alternative regulatory instructions, such as imagining standing close to the aversive stimuli (Silvers et al., 2012, study 2; Silvers et al., 2015; Silvers et al., 2017). Behavioral cognitive reappraisal effectiveness was inferred from differences in self‐reported negative affect between these conditions. Neural cognitive reappraisal effectiveness, in turn, was assessed by comparing neural responses during cognitive reappraisal with those during passive viewing, indexed by reductions in amygdala activation measured with fMRI (e.g., Silvers et al., 2015, 2017; Stephanou et al., 2016) or modulations of the late positive potential measured by electroencephalography (Van Cauwenberge et al., 2017).

From these studies, there is evidence for age‐related increases in behavioral cognitive reappraisal effectiveness (McRae, Gross, et al., 2012; Guassi Moreira et al., 2019; Silvers et al., 2012, 2015, 2017; Suksasilp et al., 2021), though not all studies indicate such effects (Ahmed et al., 2018; Nook et al., 2020; Stephanou et al., 2016). In addition, there is evidence for age‐related increases in neural cognitive reappraisal effectiveness (e.g., Silvers et al., 2015, 2017; Stephanou et al., 2016; Van Cauwenberge et al., 2017).

Several factors may explain the somewhat inconsistent findings regarding behavioral cognitive reappraisal effectiveness. In the study by Stephanou et al. (2016), the sample may have been too old to capture ongoing developmental gains, as it included participants aged 15 to 25 years (*M* = 19.91), an age range in which cognitive maturation may already plateau (Luna et al., 2004). Other studies may have been limited by small sample sizes (Ahmed et al., 2018) or by the inclusion of strategies that extend beyond cognitive reappraisal, such as problem‐solving, which may be less closely tied to age‐related cognitive maturation (Nook et al., 2020).

To address these limitations, we recruited a sufficiently large sample of adolescents aged 15–19 years, an age range in which cognitive maturation of executive functions is expected (Luna et al., 2004). Also, we only included well‐established cognitive reappraisal strategies, like positive or relativizing reappraisal (McRae, Gross, et al., 2012; Silvers et al., 2012, study 1) and detached reappraisal (McRae, Gross, et al., 2012; Silvers et al., 2012, study 2; Silvers et al., 2015; Silvers et al., 2017).

## THE CURRENT STUDY

In the present daily diary study, we investigated adolescents' day‐to‐day use of cognitive reappraisal in unpleasant and pleasant events. Our focus was on state‐level associations between cognitive reappraisal use and simultaneously reported end‐of‐day subjective well‐being, conceptualized as a composite of high positive affect, low negative affect, and high daily life satisfaction. We thereby addressed three key points largely lacking in previous research on adolescents. First, we examined reappraisal in both pleasant and unpleasant situations. Second, we used a daily diary approach, which is highly ecologically valid and enables the disentangling of within‐ and between‐person effects. Third, we considered situational variations (e.g., the amount of unpleasant/pleasant events), allowing us to examine whether cognitive reappraisal was associated with subjective well‐being beyond these contextual influences. A second focus was to investigate whether the within‐person links between the two types of cognitive reappraisal and subjective well‐being would strengthen with adolescents' age, which might reflect greater effectiveness of cognitive reappraisal.

In light of our considerations, we hypothesized that the use of cognitive reappraisal strategies during the most unpleasant event and cognitive reappraisal strategies during the most pleasant event are both positively associated with end‐of‐day subjective well‐being, individually and, importantly, when considered jointly, hence beyond each other (hypothesis H1). Furthermore, we hypothesized that the strength of both these associations would be stronger the older the adolescents (hypothesis H2). Ultimately, we conducted several exploratory analyses to gain a more comprehensive understanding of the potential associations between cognitive reappraisal in unpleasant and pleasant events and subjective well‐being. Specifically, we examined links with negative affect, positive affect, and daily life satisfaction. Considering the difference between trait‐ and state‐level variables, we examined whether hypothesis H1 might also emerge at the aggregated state level, yielding trait‐like variables.

## METHODS

### Transparency and openness

The study materials, the analytical code formulated in R syntax, and the data of our investigation are available on the Open Science Framework (OSF; https://osf.io/jd4fc/overview). As this sample has been part of a larger study by the Department of Developmental Psychology of the University of Leipzig, Germany, we did not determine the sample size by an a priori power analysis, but by recruiting a similar number of participants, as in the other samples of the larger study. We have preregistered study rationale, hypotheses, study design, measures, analytical strategy, and power analyses at the OSF (https://osf.io/sb6xj/overview). We communicated changes from the preregistered measures and conducted statistical control analyses with the original measures. Importantly, we changed the originally preregistered measure of mental health, which was a composite of low depressive symptoms and high subjective well‐being, to subjective well‐being alone, as a more clearly defined outcome measure. The Faculty of Life Sciences' Institutional Review Board at the University of Leipzig approved the larger study to which this study pertains.

In the preregistration, we conducted simulation‐based power analyses for a multilevel random intercept–random slope model, assuming small, small to moderate, and moderate effect sizes, based on the final sample size of 122 participants with 22 entries per person (Arend & Schäfer, 2019). For hypothesis H1, the power to detect a significant (level 1) effect is high, thus 0.83, even if the population effect size is small. For hypothesis H2, the power to detect a significant cross‐level interaction effect is sufficient, therefore 0.71–0.76, if the population effect is of at least moderate size. This will be taken into account when interpreting the results.

### Sample

Our sample comprised 125 adolescent participants (56% female) aged 15–19 years (*M* = 17.01, *SD* = 1.42) with approximately 25 participants per year of age, who provided 3083 valid daily diary entries (entries per person: *M* = 24.28, *SD* = 5.83; average completion rate: 86.71%). Of these, four entries were excluded due to speeding (i.e., less than 168 s). To appropriately assess end‐of‐day subjective well‐being, we excluded entries filled out after 5 am, leaving out another 314 entries. Finally, three participants were excluded because they quit the study due to a lack of motivation (they had provided two or three entries). The final sample thus consisted of 122 adolescents (57% female), aged 15–19 years (*M* = 17.00, *SD* = 1.42), with a total of 2757 entries (entries per person: *M* = 22.60, *SD* = 5.93, range: 5–28 days).

Participants were recruited in Leipzig, Germany, by advertisements posted on social media, on the street, and at showcases held at schools, training companies, and the university. Inclusion criteria were: (a) chronological age of 15–19 years, (b) full understanding of [language], (c) no holidays or extraordinary events during the daily diary, (d) informed consent of the adolescent and, for participants younger than 18 years, an informed consent of a parent with custody. We aimed to recruit a heterogeneous sample regarding socioeconomic background variables (e.g., different school types). Still, our sample of adolescents ended up with a moderately higher educational background (56% attending academic high school, 22% studying at university vs. 12% attending middle school, 8% doing a traineeship, 2% miscellaneous) than the average German adolescent population (Bund‐Länder‐Demografieportal, 2021; Statistisches Bundesamt, 2022).

### Procedure

After a parent with custody had filled out an online questionnaire and provided informed consent, participants completed cognitive performance tests and questionnaires assessing demographics (e.g., age, gender, educational background) and constructs not used in this study (for details, see Supplemental Materials 2 at https://osf.io/jd4fc/overview). Next, participants were introduced to handling the daily diary, which was hosted on Formr (Arslan et al., 2020). Daily participation reminders were sent out via e‐mail. The daily diary could be accessed via smartphone, computer, or tablet. After participants completed a daily diary under the guidance of an instructor (the first author or student assistants), they were asked to fill out the diary each evening after 8 pm and before going to bed. The daily diary assessed data in the following order: (a) Current (thus end‐of‐day) subjective well‐being; (b) emotions and emotion regulation strategies, including cognitive reappraisal, employed during the most unpleasant event of the day; (c) emotions and emotion regulation strategies, including cognitive reappraisal, employed during the most pleasant event of the day; (d) amount of unpleasant and pleasant events experienced that day. We merely excluded the few (2.9%) entries in which participants had not reported on all of these items, as is often done in daily diaries (e.g., Newman & Nezlek, 2022).

The order of presentation of all parts and items of the daily diary was kept unchanged to minimize the risk of answering incorrectly by chance. After 28 days, participants provided feedback, were debriefed and received up to 104€ or 9.5 course credits for their studies, plus 14€ as compensation. Data collection started in May 2023 and ended in January 2024.

### Measures

#### Within‐person level variables

##### End‐of‐day subjective well‐being

At the end of each day, participants began their diary entry by reporting how intensely they were currently feeling a range of eight distinct emotions and how satisfied they currently were with their day and life (i.e., daily life satisfaction). Subjective well‐being was calculated as the mean of reversed‐coded negative affect, positive affect, and daily life satisfaction (all weighted equally). Negative affect was assessed with four items (afraid, upset, guilty, irritable) of the Positive and Negative Affect Schedule (PANAS; Krohne et al., 1996; Watson & Clark, 1988) and one additional emotion item (worried), while positive affect was assessed with three additional emotion items (satisfied, optimistic, joyful). Daily life satisfaction was assessed with two items (At the current moment: Satisfied with my life, At the current moment: Satisfied with my day), based on Oishi et al. (2007). Emotions and daily life satisfaction were assessed on an 11‐point scale ranging from *not at all* (0) to *very intensely* (10). Within‐ and between‐person reliability was good for subjective well‐being and its components (see Table 1).

**TABLE 1:** Descriptive statistics of the state‐level variables and covariates.

Variables	*M* (*SD*)	Possible range	ω_within_	ω_between_	ICC
Subjective well‐being	6.67 (1.11)	0–10	.84	.84	.42
Negative affect	1.96 (1.13)	0–10	.72	.94	.49
Positive affect	5.65 (1.48)	0–10	.78	.85	.39
Daily life satisfaction	6.32 (1.33)	0–10	.63	.85	.48
Cognitive reappraisal in unpleasant events	3.89 (1.53)	0–10	.58	.85	.42
Cognitive reappraisal in pleasant events	5.14 (1.48)	0–10	.52	.88	.50
Amount of unpleasant events	3.67 (1.82)	0–10	–	.92^a^	.32
Amount of pleasant events	6.05 (1.75)	0–10	–	.88^a^	.29
Negative affect during the unpleasant event	4.39 (1.32)	0–10	.63	.92	.47
Pleasant affect during the pleasant event	6.56 (1.04)	0–10	.59	.82	.49

Abbreviation: ICC, Intraclass correlation.

^a^ Spearman‐Brown reliability coefficient, calculated by comparing the aggregated number of unpleasant/pleasant events on odd and even days.

##### Use of cognitive reappraisal strategies

Participants were asked how intensely they employed cognitive reappraisal strategies during the most unpleasant and most pleasant event of the day, with the following instruction: Please report what you have thought or done in the situation to influence your emotions. Please indicate for each of the following strategies to what degree you have used it. Below, all emotion regulation strategies were listed on 11‐point scales ranging from *not at all* (0) to *very intensely* (10). Strategies of cognitive reappraisal in unpleasant events were: *Positive reappraisal* (“I tried seeing the situation as positively as possible”), *relativizing reappraisal* (“I thought that the situation might turn out not that bad after all”), *detached reappraisal* (“I thought about the situation in a detached manner”), and *self‐assurance* (“I thought how my strengths might help me in the situation”). Positive, relativizing, and detached reappraisal were based upon an emotion regulation questionnaire (FEEL‐KJ; Grob & Smolenski, 2005) and various studies (McRae, Gross, et al., 2012; Webb et al., 2012). Self‐assurance was derived from its parallel cognitive reappraisal strategy in pleasant events, self‐reinforcement, as we aimed to create parallel strategies for cognitive reappraisal in unpleasant and pleasant events. Strategies of cognitive reappraisal in pleasant events were: *Reappraisal as more positive* (“I thought about the situation repeating itself or having positive consequences”), *reappraisal as special* (“I thought that the situation was a very special moment”), *positive similar event* (“I remembered situations which were similarly pleasant”), and *self‐reinforcement* (“I thought about how my strengths contributed to the situation”). While reappraisal as special and self‐reinforcement were based upon the Responses to Positive Affect questionnaire (RPA; Feldman et al., 2008), reappraisal as more positive and positive similar event were based on a study by Quoidbach et al. (2010). To improve their reliability, we changed the composite scores of cognitive reappraisal in unpleasant events and cognitive reappraisal in pleasant events to each encompass four items instead of three items, thus deviating from our preregistration. For cognitive reappraisal in unpleasant and pleasant events, this yielded good between‐person reliabilities (ω_between_ = .85 and ω_between_ = .88, respectively), but only acceptable within‐person reliabilities, likely due to variations in strategy use across days (ω_within_ = .58 and ω_within_ = .52, respectively). Spearman‐Brown split‐half retest‐reliabilities were good with *r*_*tt*_ ≥ .88 for each of the individual cognitive reappraisal strategies. To address the possibility that the strategies differentially covary with the outcomes, we conducted four control analyses, in which we compared a cognitive reappraisal strategy in unpleasant events with a parallel cognitive reappraisal strategy in pleasant events. Descriptives are shown in Table 1.

##### Control variables

We included the number of daily diary entries as a within‐person control variable, as affect might change with it (e.g., see Li et al., 2017). We also included the amount of both unpleasant and pleasant events that occurred that day as within‐person control variables, as experiencing fewer positive or more negative events may strengthen the link between cognitive reappraisal and subjective well‐being (Li et al., 2017; Newman & Nezlek, 2022). We assessed the latter using an 11‐point Likert scale, ranging from “very few” (0) to “very many” (10).

#### Between‐person level variables

##### Trait‐level control variables

We included gender (male, female, diverse, no answer), as it can alter which emotion regulation strategies are chosen (Chadwick et al., 2020). Participants only reported female or male gender, which we dummy‐coded. We also included the aggregated level of negative affect during the most unpleasant event of the day, assessed with seven items (afraid, upset, guilty, worried, unhappy, irritable, tense), and positive affect during the most pleasant event of the day, using six items (satisfied, relaxed, joyful, proud, grateful, optimistic). We included the latter two because they may have influenced cognitive reappraisal choice (Newman & Nezlek, 2022).

##### Aggregated state‐level variables

For preregistered exploratory analyses, we aggregated state‐level measures of subjective well‐being, cognitive reappraisal in unpleasant events, and cognitive reappraisal in pleasant events across the number of days for each person, resulting in trait‐like variables.

### Analytic strategy

To test our main hypotheses, we computed 2‐level multilevel models (MLM), in which days constitute level 1, nested within participants, who constitute level 2. To test hypothesis H1, we computed a random intercept–random slope model. Level 1 predictors were: (1) usage of cognitive reappraisal strategies in unpleasant events; (2) usage of cognitive reappraisal strategies in pleasant events. Level 1 covariates were: (1) day of the daily diary; (2) and (3) amount of unpleasant and pleasant events that day. The outcome variable was end‐of‐day subjective well‐being. Level 2 covariates were: (1) gender; (2) and (3) aggregated level of negative affect during the most negative and the most positive event of the day. For our analyses, we used a forward‐step approach: First, the level 1 analysis only included the use of cognitive reappraisal strategies in unpleasant events. Next, it only included the use of cognitive reappraisal strategies in pleasant events. Finally, it included the usage of cognitive reappraisal strategies in both pleasant and unpleasant events. We then exploratively repeated these analyses with negative affect, positive affect, and daily life satisfaction as outcome measures. Moreover, we exploratively analyzed aggregated state‐level variables using multiple regression analyses.

To test hypothesis H2, we also employed a random intercept–random slope model, in which the effect of the relevant level 1 predictors on subjective well‐being was predicted by age (a cross‐level interaction). For both analyses, we used person‐mean‐centred level 1 predictors and covariates, and grand‐mean‐centred level 2 predictors and covariates to specifically investigate variance on level 1 or level 2, respectively (see Li et al., 2017).

All analyses were conducted in R 4.3 (R Core Team, 2023), using the psych package to estimate between‐ and within‐person reliabilities and intraclass correlations (Revelle, 2024). We estimated all MLMs using the lme4 package (Bates et al., 2015), and significance tests in these models were based on the Satterthwaite degrees of freedom approximation of the lmerTest package (Kuznetsova et al., 2017). All hypothesis tests used a significance level of α = .05.

## RESULTS

### Preliminary analyses

First, we calculated bivariate correlations of the state‐level variables and the aggregated state‐level variables across the number of days (see Table 2). At this zero‐order level, cognitive reappraisal in unpleasant events and cognitive reappraisal in pleasant events showed similar correlations with subjective well‐being at the state level, with the latter consistently displaying greater associations with subjective well‐being and its components. Furthermore, the state‐level correlations with subjective well‐being were mainly driven by associations with positive affect and daily life satisfaction. At the aggregated state level, cognitive reappraisal in unpleasant events and cognitive reappraisal in pleasant events showed similar associations with subjective well‐being, driven mainly by positive affect. Cognitive reappraisal in unpleasant events and pleasant events were only slightly correlated on a state level (*r*(2755) = .15, *p* < .001), but displayed much greater correlations on an aggregated state level (*r*(120) = .63, *p* < .001).

**TABLE 2:** Pearsons' product moment correlations between the main state‐level and aggregated state‐level variables.

Variables	1		2		3		4		5	
	*r*_state_	*r*_agrr. state_	*r*_state_	*r*_agrr. state_	*r*_state_	*r*_agrr. state_	*r*_state_	*r*_agrr. state_	*r*_state_	*r*_agrr. state_
1. Subjective well‐being										
2. Negative affect	−.76***	−.61***								
3. Positive affect	.89***	.80***	−.51***	−.10						
4. Daily life satisfaction	.86***	.92***	−.48***	−.38***	.67***	.74***				
5. Cognitive reappraisal in unpleasant events	.19***	.28**	−.10***	.17	.19***	.54***	.17***	.26**		
6. Cognitive reappraisal in pleasant events	.25***	.24**	−.11***	.28**	.25***	.56***	.25***	.25**	.15***	.63***

*Note*: State‐level variables reflect the momentary day of an individual, and the correlations indicate within‐person associations. Aggregated state‐level variables are state‐level variables aggregated across the number of days. They are akin to trait‐level data, and the correlations reflect between‐person associations. *p < .05, **p < .01, ***p < .001.

### H1: Associations beyond another of cognitive reappraisals and subjective well‐being

Cognitive reappraisal in unpleasant events was significantly associated with end‐of‐day subjective well‐being, *b* = 0.10, 95% CI = [0.07, 0.13], *SE* = .02, *t*(124.47) = 6.88, *p* < .001, *d* = 1.23, and so was cognitive reappraisal in pleasant events, *b* = .10, 95% CI = [0.07, 0.13], *SE* = .02, *t*(95.66) = 6.34, *p* <. 001, *d* = 1.30. Importantly, and consistent with our first prediction, when specified jointly as predictors of subjective well‐being, both cognitive reappraisal in unpleasant events and cognitive reappraisal in pleasant events still showed very similar associations of *b* = 0.08, 95% CI = [0.05, 0.10], *SE* = .01, *t*(120.20) = 5.45, *p* < .001, *d* = 0.99, and *b* = 0.08, 95% CI = [0.06, 0.11], *SE* = .01, *t*(103.14) = 5.82, *p* < .001, *d* = 1.15, respectively.

Follow‐up analyses with the components of subjective well‐being as outcomes revealed distinct patterns: When considered jointly, associations with negative affect emerged only for cognitive reappraisal in unpleasant events (*p* = .012, *d* = 0.50), but not for cognitive reappraisal in pleasant events (*p* = .313, *d* = 0.22). Regarding positive affect and daily life satisfaction, joint analyses showed significant associations with both cognitive reappraisal in unpleasant events (*p* < .001, *d* = 1.05 and *p* < .001, *d* = 0.84, respectively) and cognitive reappraisal in pleasant events (*p* < .001, *d* = 1.40 and *p* < .001, *d* = 0.97, respectively). For more details on these analyses, see Chapter S1, and for associations of more emotion regulation strategies with subjective well‐being, see Table S1 in Chapter S3 of Supplemental Materials S1.

#### Statistical control analyses regarding H1

To adhere to the slightly different predictors we had preregistered, we repeated all analyses with cognitive reappraisal in unpleasant and pleasant events, with three instead of four strategies. The joint analysis of subjective well‐being with cognitive reappraisal in unpleasant events and in pleasant events remained significant, with respectively *p* < .001, *d* = 0.97 and *p* < .001, *d* = 1.22. In light of the only moderate within‐person reliabilities of cognitive reappraisal in unpleasant and pleasant events, we also conducted four multilevel models in which we pitched a cognitive reappraisal strategy in unpleasant events against a parallel cognitive reappraisal strategy in pleasant events. We compared positive reappraisal with reappraisal for positive outcomes, relativizing reappraisal with reappraisal as special, self‐assurance with self‐reinforcement, and detached reappraisal with positive similar event. Significance levels ranged from *p* < .05 to *p* < .001 and effect sizes from *d* = 0.45 to *d* = 0.98. In sum, none of the statistical control analyses changed the results (for details, see Supplemental Materials S1, Chapter S1).

#### Associations of cognitive reappraisal and subjective well‐being on a between‐person level

We additionally conducted linear models using the aggregated state‐level variables. We computed three linear models: one including cognitive reappraisal in unpleasant events, another cognitive reappraisal in pleasant events, and a third including cognitive reappraisal in unpleasant and pleasant events. For each of these models, covariates were the average number of days of the daily diary, the average amount of unpleasant and pleasant events, and the participants' gender. For aggregated state‐level data, cognitive reappraisal in unpleasant events was significantly related to average subjective well‐being, *b* = 0.14, 95% CI = [0.04, 0.25], *SE* = .05, *t*(116) = 2.63, *p* < .01, *d* = 0.49, and so was cognitive reappraisal in pleasant events, *b* = 0.12, 95% CI = [0.02, 0.21], *SE* = .05, *t*(116) = 2.49, *p* < .05, *d* = 0.46. However, once aggregated state‐level cognitive reappraisal in unpleasant events and cognitive reappraisal in pleasant events were included, neither displayed significant associations with subjective well‐being any longer, *b* = 0.10, 95% CI = [−0.04, 0.23], *SE* = .07, *t*(115) = 1.42, *p* = .158, *d* = 0.27, and *b* = 0.07, 95% CI = [−0.05, 0.19], *SE* = .06, *t*(115) = 1.15, *p* = .254, *d* = 0.21, respectively. In light of the high correlation between both cognitive reappraisal types (*r* = .63), we exploratively examined the association of a composite of cognitive reappraisal in unpleasant and pleasant events and subjective well‐being. A significant link emerged of *b* = 0.16, 95% CI = [0.05,0.27], *SE* = .06, *t*(116) = 2.88, *p* < .01, *d* = 0.53 (see Supplemental Materials S1, Chapter S1).

### H2: Associations of cognitive reappraisals and subjective well‐being: The role of adolescents' chronological age

In contrast to our predictions, both the cross‐level interactions of age with cognitive reappraisal in unpleasant events and of age with cognitive reappraisal in pleasant events were nonsignificant, *b* = 0.00, 95% CI = [−0.02, 0.02], *SE* = .01, *t*(108.36) = 0.51, *p* = .863, *d* = 0.03, and *b* = −0.02, 95% CI = [−0.04, 0.00], *SE* = .01, *t*(97.33) = −1.81, *p* = .073, *d* = 0.37, respectively. Non‐preregistered follow‐up analyses with each of the four cognitive reappraisal strategies in unpleasant and pleasant events indicated no cross‐level interactions (all strategies *p* > .064).

#### Statistical control analyses regarding H2

Finally, the results remained unchanged when we reran the analyses with the preregistered predictors (see Supplemental Materials S1, Chapter S2).

## DISCUSSION

This daily diary study is the first to examine if cognitive reappraisal in unpleasant events and cognitive reappraisal in pleasant events are both associated not just individually, but also beyond each other with adolescents' end‐of‐day subjective well‐being, being composed of low negative affect, high positive affect and high daily life satisfaction. Accounting for adolescents' rapid neuronal maturation (Keshavan et al., 2014), we also examined whether the associations of cognitive reappraisal in unpleasant and pleasant events and subjective well‐being are moderated by adolescents' age (Ochsner & Gross, 2005). As predicted, adolescents' end‐of‐day subjective well‐being was higher on days on which they employed cognitive reappraisal more than usual, both during the day's most unpleasant and the most pleasant event. Importantly, both types of cognitive reappraisal were linked to higher end‐of‐day subjective well‐being beyond each other. Consequently, neither cognitive reappraisal in unpleasant events nor cognitive reappraisal in pleasant events is “redundant”, as both have their own, unique relations to subjective well‐being. Hence, both cognitive reappraisal in unpleasant events and in pleasant events are closely linked to adolescents' subjective well‐being.

Interestingly, not only cognitive reappraisal in pleasant events, but also cognitive reappraisal in unpleasant events, thus mostly directed at unpleasant emotions, was associated with higher positive affect and daily life satisfaction. This means that reappraising unpleasant events is not only associated with lower negative affect, but also, either indirectly via lower negative affect or even directly, with more of the positive components of subjective well‐being. Contrary to the within‐person findings, the between‐person analyses suggested that aggregated state‐level cognitive reappraisals in unpleasant and pleasant events were no longer associated with subjective well‐being once considered simultaneously. Finally, inconsistent with our second hypothesis, age did not moderate the associations of cognitive reappraisal and subjective well‐being. All results remained unchanged in the statistical control analyses.

### The associations of both types of cognitive reappraisals with subjective well‐being

Our study is the first to investigate cognitive reappraisals in both types of events and consider their joint associations with subjective well‐being. Notably, at the aggregated state level, cognitive reappraisal in both unpleasant and pleasant events was only associated with higher subjective well‐being when considered alone, but not when considered jointly. Why do these findings differ from those of the state variables? A likely explanation is the vast difference in shared variance among predictors: While cognitive reappraisal in unpleasant and pleasant events were only correlated at *r* = .15 on a state level, correlations on the aggregated state were much greater (*r* = .63). This greater shared variance led to multicollinearity and left less unique variance for cognitive reappraisal in unpleasant and in pleasant events. Thus, when considered jointly, no significant association remained at the aggregated state level. This makes interpreting between‐level results more difficult. Importantly, a composite of cognitive reappraisal in both unpleasant and pleasant events was significantly associated with subjective well‐being, likely as multicollinearity was avoided.

Conceptually, the low correlations on a within‐person level signify that on days during which adolescents use cognitive reappraisal in the most unpleasant event, they are only slightly more likely to also use cognitive reappraisal in the most pleasant event that day. High correlations at the aggregated state level mean that there is an overall tendency to reappraise, both in pleasant and in unpleasant events. In comparison, the much lower correlations at the state level mean that on days on which adolescents reappraise pleasant events, they do not necessarily reappraise unpleasant events, and vice versa. Considering this discrepancy, adolescents may employ reappraisal in response to situational affordances (e.g., perceived controllability of an event; Wenzel et al., 2019), rather than as a habitual response to pleasant and unpleasant situations. Thus, future studies should investigate how situational circumstances are associated with adolescents' choice of emotion regulation in unpleasant and pleasant events.

Finally, these results also suggest that considering only between‐person associations might lead to the incorrect conclusion that both types of reappraisal do not have predictive value above and beyond one another regarding subjective well‐being. However, while adolescents who use cognitive reappraisal in unpleasant situations may also be more likely to use reappraisal in pleasant situations, the reappraisal types are used in various situations, potentially with different affordances, both contributing to everyday subjective well‐being.

### Age as moderator of the association of cognitive reappraisal and subjective well‐being

Unexpectedly, cognitive reappraisal strategies in unpleasant and pleasant events, both as a composite and as individual strategies, failed to exhibit stronger associations with subjective well‐being as the adolescents grew older. This might be due to three factors:

The first might be the use of a daily diary instead of a laboratory study. In laboratory studies, participants had 30 to 60 trials in short succession in which they had to use cognitive reappraisal (e.g., McRae, Gross, et al., 2012; Silvers et al., 2012, 2015, 2017). As adolescents' rapidly maturing cognitive abilities (Luna et al., 2004) also facilitate learning per se, experimental studies may have measured a stronger training effect in older adolescents, but not improvements in cognitive reappraisal effectiveness. In contrast, in our daily diary study, adolescents reported using several different emotion regulation strategies daily, making training effects unlikely.

Second, it is possible that age‐related differences are not driven by improvements in executive functioning, as proposed by Ochsner and Gross (2005). This would have affected most of our cognitive reappraisal strategies, as those focused on reappraising stimuli (such as positive reappraisal) are especially closely linked to executive functioning (Messina et al., 2015). Perhaps it is not only improvements in executive functioning, but also enhanced neural connectivity (Keshavan et al., 2014) that underlie greater cognitive reappraisal effectiveness.

Third, instead of cross‐sectional age comparison, longitudinal changes in age‐related cognitive gains or underlying brain maturation (over months or years) might have displayed stronger associations with longitudinal variation in cognitive reappraisal effectiveness. Consequently, researchers could conduct laboratory studies that account for putative training effects or combine different research designs, such as a laboratory and a daily diary study. This would help determine whether a training effect underlies age‐related differences in cognitive reappraisal effectiveness, and whether findings differ on a trait‐level compared to a state level. Such studies could be further improved by including repeated measurements to capture age‐related cognitive maturation processes on a within‐person level, which would allow for testing of associations of subjective well‐being and cognitive gains on a within‐person level.

### Limitations and future directions

As with any study, ours also has limitations, which will hopefully lead to future research. The first limitation is our WEIRD (White, Educated, Industrialized, Rich, Democratic) sample. Though most studies in adults (e.g., Heiy & Cheavens, 2014; Newman & Nezlek, 2022) and adolescents (e.g., Chadwick et al., 2020; Verzeletti et al., 2016) have been conducted in Western countries, including our study taking place in Germany, emotion regulation might differ in other cultures. As cultural socialization shapes adolescents' developing sense of self and their relation to emotions of oneself and others (Keller & Joscha, 2013), the use and effectiveness of emotion regulation strategies vary across cultures. For example, an expression of feelings might serve to display one's identity (in cultures valuing independence) or facilitate relatedness (in cultures valuing relatedness; Keller & Joscha, 2013). The emotion regulation strategy we are focusing on here, cognitive reappraisal, seems to exhibit fewer cross‐cultural differences in effectiveness (Hu et al., 2014), but stark cross‐cultural variations in its usage may be expected. A second limitation is that emotions and moods can impact the evaluation of current or former states, especially if they are less memorable (Schwarz et al., 1987; Shiffman et al., 2008). Hence, memory biases might have influenced reports on cognitive reappraisal and affect during the most unpleasant and most pleasant event of the day. For example, low subjective well‐being at the end of the day may have led to a negatively biased rating of the events during the day and the reappraisal use during these events—to decrease such a bias, we did not include state‐level affect in the analyses. Additionally, recent cognitive reappraisal just before the daily diary may have led to an overestimation of former cognitive reappraisal use (Shiffman et al., 2008). Other research designs might further reduce biases, such as ecological momentary assessment. The third limitation concerns our sample and choice of materials: Our sample was of a higher educational background, and thus likely also of a higher socioeconomic status, than the general [nationality] adolescent population. Previous research has indicated that cognitive reappraisal is more strongly related to lower depressive symptoms in individuals of lower rather than higher socioeconomic status (Troy et al., 2017). Thus, associations between reappraisal and subjective well‐being might be stronger in a more diverse sample. Future studies may investigate the role of situational characteristics (e.g., perceived controllability; Wenzel et al., 2019) and socioeconomic status in emotion regulation strategy use and effectiveness. A fourth limiting factor is that our results are correlational; thus, no causal inferences may be drawn. Future laboratory studies would be required for drawing any causal inferences.

### Practical implications

Adolescents face significant challenges in regulating their emotions in response to both unpleasant and pleasant events—and at the same time, emotion regulation might be highly malleable at this developmental stage (Gilbert, 2012). It would thus be important and effective to offer adolescents interventions that help them improve their emotion‐regulation skills. Considering our purely correlational results, future studies on cognitive reappraisal training (e.g., Kam et al., 2024) might want to investigate whether emotion regulation programs would be more effective in improving adolescents' subjective well‐being if they were to foster effective emotion regulation in unpleasant and pleasant events.

Furthermore, our findings suggest that adolescence is not just a phase of “storm and stress” (Hall, 1904), with adolescents only experiencing greater affective instability (Reitsema et al., 2022), but that it is also shaped by pleasant events, pleasant emotions and their regulation. Adolescence brings the emergence of several new pleasant events, such as profound friendships (Rice & Mulkeen, 1995) and romantic relationships (Zimmer‐Gembeck, 1999). Consistently, this study and former studies suggest that, in their daily lives, adolescents experience higher positive affect than negative affect (e.g., Ronen et al., 2016). Ultimately, our results suggest that not just cognitive reappraisal in unpleasant events, but also cognitive reappraisal in pleasant events, are associated with higher end‐of‐day subjective well‐being, individually and beyond each other. Only by acknowledging the importance of pleasant events, pleasant emotions and their regulation in adolescents' lives can we optimally support them in their development.

## CONCLUSION

In the present daily diary study, we investigated whether and how cognitive reappraisal in unpleasant events and cognitive reappraisal in pleasant events are related to adolescents' end‐of‐day subjective well‐being, and whether adolescents' age would moderate these associations. Our results suggest that cognitive reappraisal in unpleasant events and cognitive reappraisal in pleasant events are both associated with adolescents' subjective well‐being beyond each other.

Our results show that associations of reappraisal and subjective well‐being are similar across adolescents aged 15–19 years. This could suggest that they can use cognitive reappraisal strategies in similar ways. Finally, while on average, adolescents who used reappraisal in unpleasant events tended to use it in pleasant events as well, on a day‐to‐day basis, they seemed to choose their emotion regulation strategies according to situational characteristics. To further our understanding of how adolescents regulate their emotions, we must consider the situational characteristics of the unpleasant and pleasant events they encounter, as these may significantly influence whether adolescents employ cognitive reappraisal strategies effectively. To conclude, our research findings suggest that not only adolescents' cognitive reappraisal in unpleasant events but also their cognitive reappraisal in pleasant events is important for their subjective well‐being. Moving forward, to gain a more comprehensive understanding of adolescents' emotion regulation, future studies may investigate emotion regulation in both unpleasant and pleasant events, while also accounting for situational characteristics.

## AUTHOR CONTRIBUTIONS

This study was conceptualized by Felix Sternke, Ute Kunzmann, and Elisabeth S. Blanke and conducted and analyzed by Felix Sternke. The paper was written by Felix Sternke, with support and supervision by Ute Kunzmann, Steffen Nestler, and Elisabeth S. Blanke. Furthermore, Ute Kunzmann provided supervision for setting up the study, while Steffen Nestler supervised the data analysis, and Elisabeth S. Blanke supervised the validation of findings.

## FUNDING INFORMATION

This paper is part of an overarching research project (ID: 451942112) funded by the German Research Foundation (grant number 451942112), which has been awarded to Ute Kunzmann (KU1267/12‐1), Steffen Nestler (NE1485/9‐1), and Denis Gerstorf (GE1896/8‐1) in cooperation with Carsten Wrosch.

## CONFLICT OF INTEREST STATEMENT

We do not have any conflicts of interest to declare.

## ETHICS STATEMENT

The larger project was approved on the 23rd of September 2020 by the Faculty of Life Sciences' Institutional Review Board of the University of Leipzig, case number 2020.06.1_eb_58.

## POSITIONALITY STATEMENT

All authors are white, highly educated Germans. We are aware of our privileges and tried to recruit a heterogeneous sample regarding socioeconomic status to allow for better representation and generalization. The first author interacted with the participants; a structured interview and focus on not influencing adolescents may have improved objectivity.

## PATIENT CONSENT STATEMENT

All participants signed an informed consent form. For participants younger than 18 years, we additionally asked a parent with custody for informed consent for their child.

## Supporting information


Data S1:

Data S2:

## Data Availability

A list of all questionnaires, all items of the daily diary, the data, the R Code, and supplementary materials are at the Open Science Framework at https://osf.io/jd4fc/?view_only=47d8f7ca190c4141b086d8bbe6701ed3, reference number jd4fc. They will be openly available after acceptance.

## Footnotes

^1^
We refer to events and emotions as unpleasant and pleasant instead of negative and positive, as both are never negative and positive per se. For example, unpleasant events might entail positive consequences (e.g., negative feedback can lead to improved skills) and unpleasant emotions with their posited action tendencies (see e.g., Smith & Lazarus, 1990) might be beneficial to the long‐term well‐being (e.g., running away from a threat to survive). On the other hand, pleasant events and pleasant emotions tied to them might also entail negative consequences (e.g., talking too much about one's own achievements could lead to losing friendships).
