# Supplementary material for: Dealing with feelings in adolescence: Cognitive reappraisals in unpleasant and pleasant emotional events and their associations with subjective well‐being
Source: J Res Adolesc. 2026 Feb 25;36(1):e70162. doi: 10.1111/jora.70162 (PMC12936276; doi:10.1111/jora.70162)
Supplement: Supplementary file 1 — Data S1: [file JORA-36-0-s001.docx]

**Supplemental Materials for:**

Dealing With Feelings in Adolescence: Cognitive Reappraisals in Unpleasant and Pleasant Emotional Events and Their Associations with Subjective Well-Being

Sternke, F.^1^; Nestler, S.^2^; Blanke, E.S.^1^; Kunzmann, U.^1^

^1^ Wilhelm Wundt Institute for Psychology, Lifespan Psychology Lab, University of Leipzig

^2^ Institute for Psychology, Statistics and Psychological Methods Working Unit, University of Münster

**Author Note**

Felix Sternke
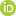
 <https://orcid.org/0000-0002-5132-1854>

Steffen Nestler
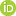
 <https://orcid.org/0000-0001-9724-2441>

Elisabeth S. Blanke
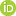
 <https://orcid.org/0000-0003-4662-1366>

Ute Kunzmann
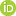
 <https://orcid.org/0000-0002-0943-7845>

Correspondence concerning this supplement should be addressed to Felix Sternke, Wilhelm Wundt Institute for Psychology, Lifespan Psychology Lab, University of Leipzig, Neumarkt 9-19, 04109 Leipzig, Germany. Email: felix.sternke@uni-leipzig.de

Directory

[Chapter S1 3](#_Toc221705563)

[More Details About the Statistical Analyses of Hypothesis H1 3](#_Toc221705564)

[Analyses Regarding the Different Components of Subjective Well-Being 3](#_Toc221705565)

[Statistical Control Analyses Regarding H1 3](#_Toc221705566)

[Associations of Cognitive Reappraisal and Subjective Well-Being on a Between-Person Level 4](#_Toc221705567)

[Chapter S2 5](#_Toc221705568)

[More Details About the Statistical Analyses of Hypothesis H2 5](#_Toc221705569)

[Statistical Control Analyses Regarding H2 5](#_Toc221705570)

[Chapter S3 6](#_Toc221705571)

[Associations of all Emotion Regulation Strategies With Subjective Well-Being 6](#_Toc221705572)

# Chapter S1

## More Details About the Statistical Analyses of Hypothesis H1

### Analyses Regarding the Different Components of Subjective Well-Being

When considered jointly, associations of negative affect with cognitive reappraisal in unpleasant events were significant, *b* = -0.04, 95% CI = [-0.07,-0.01], *SE* = .02, *t*(105.11) = -2.56, *p* < .05, *d* = 0.50, while cognitive reappraisal in pleasant events displayed non-significant associations, *b* = -0.02, 95% CI = [-0.06,0.02], *SE* = .02, *t*(87.71) = -1.01, *p* = .313, *d* = 0.22. For positive affect, when considered jointly, associations of both cognitive reappraisal in unpleasant events and cognitive reappraisal in pleasant events were significant, with *b* = 0.11, 95% CI = [0.07,0.14], *SE* = .02, *t*(128.38) = 5.92, *p* < .001, *d* = 1.05 and *b* = 0.14, 95% CI = [0.10,0.18], *SE* = .02, *t*(104.47) = 7.18, *p* < .001, *d* = 1.40, respectively. A similar pattern emerged for the associations of daily life satisfaction with cognitive reappraisal in unpleasant events and cognitive reappraisal in pleasant events, of *b* = 0.08, 95% CI = [0.04,0.11], *SE* = .02, *t*(112.24) = 4.45, *p* < .001, *d* = 0.84 and *b* = 0.09, 95% CI = [0.06,0.13], *SE* = .02, *t*(109.19) = 5.06, *p* < .001, *d* = 0.97, respectively. For associations of more emotion regulation strategies with subjective well-being, see Chapter S3 of Supplemental Materials 1.

### Statistical Control Analyses Regarding H1

We conducted the analyses with the composite scores for cognitive reappraisal in unpleasant events in pleasant events having three items each (instead of four). These were the results of the multilevel models: Cognitive reappraisal in unpleasant events and subjective well-being showed the following association: *b* = 0.08, 95% CI = [0.05,0.10], *SE* = .01, *t*(121.52) = 5.79, *p* <.001, *d* = 1.05. Furthermore, cognitive reappraisal in pleasant events and subjective well-being had the following association: *b* = 0.09, 95% CI = [0.06,0.11], *SE* = .01, *t*(106.35) = 6.90, *p* <.001, *d* = 1.34. Finally, associations of subjective well-being with cognitive

reappraisal in unpleasant events and cognitive reappraisal in pleasant events when both were considered jointly were *b* = 0.07, 95% CI = [0.04,0.09], *SE* = .01, *t*(122.09) = 5.35, *p* <.001, *d* = 0.97, and *b* = 0.08, 95% CI = [0.05,0.10], *SE* = .01, *t*(103.74) = 6.19, *p* <.001, *d* = 1.22, respectively.

Ultimately, as the composites of cognitive reappraisal in unpleasant events and cognitive reappraisal in pleasant events had only moderate within-person reliabilities, but its individual cognitive reappraisal strategies had high split-half reliabilities, we pitched a cognitive reappraisal strategy of unpleasant emotions against a parallel cognitive reappraisal strategy of unpleasant emotions. In the multilevel model that considered the associations of subjective well-being with ‘positive reappraisal’ and ‘reappraisal for positive outcomes’, the associations were *b* = 0.05, 95% CI = [0.03,0.07], *SE* = .01, *t*(109.93) = 5.00, *p* < .001, *d* = 0.96, and *b* = 0.04, 95% CI = [0.02,0.06], *SE* = .01, *t*(74.30) = 4.00, *p* < .001, *d* = 0.93, respectively. In the multilevel model that considered the associations of subjective well-being with ‘relativizing reappraisal’ and ‘reappraisal as special’, the associations were *b* = 0.04, 95% CI = [0.02,0.06], *SE* = .01, *t*(119.26) = 4.39, *p* <.001, *d* = 0.80, and *b* = 0.05, 95% CI = [0.03,0.07], *SE* = .01, *t*(123.85) = 5.45, *p* < .001, *d* = 0.98, respectively. In the multilevel model that considered the associations of subjective well-being with ‘self-assurance’ and ‘self-reinforcement’, the associations were *b* = 0.03, 95% CI = [0.01,0.05], *SE* = .01, *t*(96.62) = 2.79, *p* <.01, *d* = 0.57, and *b* = 0.03, 95% CI = [0.01,0.05], *SE* = .01, *t*(96.55) = 3.52, *p* < .001, *d* = 0.72, respectively. Finally, in the multilevel model that considered the associations of subjective well-being with ‘detached reappraisal’ and ‘positive similar event’, the associations were *b* = 0.02, 95% CI = [0.00,0.04], *SE* = .01, *t*(113.29) = 2.39, *p* < .05, *d* = 0.45, and *b* = 0.03, 95% CI = [0.01,0.04], *SE* = .01, *t*(96.36) = 2.66, *p* < .01, *d* = 0.54, respectively.

### Associations of Cognitive Reappraisal and Subjective Well-Being on a Between-Person Level

For the multiple regressions that included aggregated state-level variables, thus state level variables aggregated over the number of days, the results were the following: cognitive reappraisal in unpleasant events and subjective well-being showed the following association: *b* = 0.14, 95% CI =[0.04,0.25], *SE* = .05, *t*(116) = 2.63, *p* < .01, *d* = 0.49. Furthermore, cognitive reappraisal in pleasant events and subjective well-being had the following association: *b* = 0.12, 95% CI = [0.02,0.21], *SE* = .05, *t*(116) = 2.50, *p* < .05, *d* = 0.46. Finally, associations of subjective well-being with cognitive reappraisal in unpleasant events and cognitive reappraisal in pleasant events when both were considered jointly were both non-significant, with *b* = 0.10, 95% CI =[-0.04,0.23], *SE* = .07, *t*(115) = 1.42, *p* = .158, *d* = 0.27, and *b* = 0.07, 95% CI = [-0.05,0.19], *SE* = .06, *t*(115) = 1.15, *p* = .254, *d* = 0.21, respectively.

Finally, considering the issue of multicollinearity between cognitive reappraisal in unpleasant events and cognitive reappraisal in pleasant events (*r* = .63), we conducted an additional exploratory analysis: We aggregated cognitive reappraisal in unpleasant events and cognitive reappraisal in pleasant events into a single aggregated-state composite score and investigated the association with subjective well-being. This analysis led to the following significant association with subjective well-being: *b* = 0.16, 95% CI = [0.05,0.27], *SE* = .06, *t*(116) = 2.88, *p* < .01, *d* = 0.53.

#

# Chapter S2

## More Details About the Statistical Analyses of Hypothesis H2

### Statistical Control Analyses Regarding H2

We reran the multilevel model with cross-level interactions with the slightly different predictors we had preregistered. Thus, cognitive reappraisal in unpleasant and pleasant events encompassed three strategies (instead of four). Both cross-level interactions stayed non-significant for cognitive reappraisal in unpleasant events with age, *b* = 0.00, 95% CI =[-0.02,0.02], *SE* = .01, *t*(110.40) = 0.18, *p* = .855, *d* = 0.03, and for cognitive reappraisal in pleasant events with age, *b* = -0.02, 95% CI = [-0.04,0.00], *SE* = .01, *t*(96.20) = -1.72, *p* = .089, *d* = 0.35.

# Chapter S3

## Associations of all Emotion Regulation Strategies With Subjective Well-Being

**Table S1**

*Associations of all Emotion Regulation Strategies With Within-Person Subjective Well-Being*

| Emotion regulation strategies in unpleasant events | | | | | | | Emotion regulation strategies in pleasant events | | | | | |  |
| --- | --- | --- | --- | --- | --- | --- | --- | --- | --- | --- | --- | --- | --- |
| Strategy name | *b* | 95% CI | | *p* | | *d* | | Strategy name | Est. | 95% CI | *p* | *d* |  |
| Positive reappraisal | 0.05 | [0.03, 0.07] | | <.001 | | 0.97 | | Reappraisal for positive outcomes | 0.04 | [0.02, 0.06] | <.001 | 0.98 | |
| Relativizing reappraisal | 0.04 | [0.03, 0.06] | | <.01 | | 0.83 | | Reappraisal as special | 0.05 | [0.04, 0.07] | <.001 | 1.01 | |
| Self-assurance | 0.03 | [0.01, 0.05] | | <.001 | | 0.66 | | Self-reinforcement | 0.04 | [0.02, 0.05] | <.001 | 0.77 | |
| Detached reappraisal | 0.02 | [0.00, 0.04] | | <.05 | | 0.44 | | Positive similar event | 0.02 | [0.01, 0.04] | <.05 | 0.53 | |
| Distraction | 0.01 | [-0.01, 0.02] | | .435 | | 0.03 | | Enjoying the moment | 0.07 | [0.05, 0.09] | <.001 | 1.53 | |
| Directing attention | -0.01 | [-0.03, 0.01] | | .381 | | 0.16 | | Directing attention | 0.06 | [0.04, 0.08] | <.001 | 0.99 | |
| Catastrophizing | -0.03 | [-0.05, -0.01] | | <.01 | | 0.66 | | Attention on negatives | -0.03 | [-0.06, -0.01] | <.05 | 0.63 | |
| Expression suppression | 0.03 | [0.01, 0.05] | | <.001 | | 0.99 | | Distraction | -0.04 | [-0.06, -0.02] | <.01 | 0.73 | |
| Situation modification | 0.04 | [0.02, 0.05] | | <.001 | | 0.59 | | Situation modification | 0.05 | [0.03, 0.07] | <.001 | 1.10 | |
| Problem-solving | 0.02 | [0.00, 0.03] | | <.05 | | 0.41 | | Expression of emotions | 0.05 | [0.03, 0.07] | <.001 | 1.26 | |
|  |  |  |  | |  | | | Social sharing | 0.02 | [-0.00, 0.03] | .079 | 0.32 | |
| *Note.* Findings of separate 2-level multilevel random-slope random-intercept models examining the associations of individual emotion regulation strategies with end-of-day subjective well-being. They contained the same covariates as the analyses of hypothesis H1. Est. = estimate, CI = confidence interval. Positive values indicate greater associations. | | | | | | | | | | | | |  |
