# Supplementary material for: Dealing with feelings in adolescence: Cognitive reappraisals in unpleasant and pleasant emotional events and their associations with subjective well‐being
Source: J Res Adolesc. 2026 Feb 25;36(1):e70162. doi: 10.1111/jora.70162 (PMC12936276; doi:10.1111/jora.70162)
Supplement: Supplementary file 2 — Data S2: [file JORA-36-0-s002.docx]

**Detailed Description of all Parts of the Study:**

Dealing With Feelings in Adolescence: Cognitive Reappraisals in Unpleasant and Pleasant Emotional Events and Their Associations with Subjective Well-Being

Sternke, F.^1^; Nestler, S.^2^; Blanke, E.S.^1^; Kunzmann, U.^1^

^1^ Wilhelm Wundt Institute for Psychology, Lifespan Psychology Lab, University of Leipzig

^2^ Institute for Psychology, Statistics and Psychological Methods Working Unit, University of Münster

**Author Note**

Felix Sternke
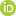
 <https://orcid.org/0000-0002-5132-1854>

Steffen Nestler
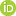
 <https://orcid.org/0000-0001-9724-2441>

Elisabeth S. Blanke
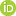
 <https://orcid.org/0000-0003-4662-1366>

Ute Kunzmann
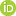
 <https://orcid.org/0000-0002-0943-7845>

Correspondence concerning this supplement should be addressed to Felix Sternke, Wilhelm Wundt Institute for Psychology, Lifespan Psychology Lab, University of Leipzig, Neumarkt 9-19, 04109 Leipzig, Germany. Email: felix.sternke@uni-leipzig.

Directory

[Detailing the Full Scope of the Study 3](#_Toc221704798)

[Online Questionnaire Filled out by a Parent 3](#_Toc221704799)

[Introductory Session 7](#_Toc221704800)

[Daily Diary 12](#_Toc221704801)

[Debriefing 24](#_Toc221704802)

[References 27](#_Toc221704803)

# Detailing the Full Scope of the Study

This study is part of a research project containing five different elements, forming part of an overarching research project funded by the National Research Association of Germany (ID: 451942112). This overarching research project investigated age differences in emotion regulation flexibility, more specifically, emotion regulation that shows a certain strategy-situation fit. The study investigated age differences between younger adults (20-30 years old) and older adults (65-80 years old), while the part of the study which investigated emotion regulation in adolescents (15-19 years old) was added later on. While the overarching study encompassed many components, such as an introductory session, a daily diary study (28 days), a laboratory study, an ecological momentary assessment study (9 days) and a debriefing, the part of the study investigating emotion regulation in adolescents consisted only of an introductory session, a daily diary study (28 days), and a debriefing session. The overarching study was approved by the ethics advisory board of the University of Leipzig, Germany.

To go into further detail regarding the study about adolescents, which the paper is based on, it should be emphasised that interactions with participants were kept highly structured by an interview and rigorous training of experimenters.

## Online Questionnaire Filled out by a Parent

1. Participants were only allowed to partake in the study if a parent had filled out a set of questionnaires before the adolescents’ introductory session. These questionnaires were also hosted on the platform formr 0.19.4 and 0.20.6 (Arslan et al., 2020).
2. On the first page, parents had to provide informed consent by reading through the terms of the study and accepting them by clicking the respective field.
3. They were then required to enter a certain code as an identifier for their child, which later on allowed us to match adolescent and parental data.
4. Next, parents provided information about their socioeconomic status. Among them were age, gender (female, male, other, I don’t want to respond), the highest level of education they have achieved, profession, net income, household income, persons living in the family, formal family status (for example, married or divorced), and who they lived with in the same household.
5. Then the parents were asked to fill out the following questionnaires:
   1. A single item assessing life satisfaction (SOEP Group, 2023).
   2. An abbreviated version of the Positive and Negative Affect Schedule (Krohne et al., 1996; Watson & Clark, 1988), 17 items, eight additional items assessing positive and negative affect, as well as the five items for assessing depressive symptoms.
   3. A newly created inventory of 10 strategies for emotion regulation in unpleasant events. This was the same as for the parent. These were the same as in the daily diary. Participants were asked on an 11-point scale ranging from 0 (never) to 0 (extremely often) how often they were performing these activities or thoughts:
   4. I contemplate how I could solve the problem. (Problem-solving)
   5. I think about how my strengths could help me in the situation. (Self-assurance, thus a cognitive reappraisal (cognitive reappraisal) strategy)
   6. I try viewing the situation in a more positive light. (Positive reappraisal, thus a cognitive reappraisal strategy)
   7. I think that the situation might not turn out that bad. (Relativizing reappraisal, thus a cognitive reappraisal strategy)
   8. I get active and try changing the situation for the better. (Situation modification)
   9. I distract myself and direct my attention to other things. (Distraction)
   10. I do not let my unpleasant emotions show. (Expression suppression)
   11. I think about the situation in a detached manner. (Detached reappraisal, thus a cognitive reappraisal strategy)
   12. I imagine how bad the situation might still get. (Catastrophizing)
   13. I pay full attention to the situation. (Directing attention)
   14. A newly created inventory of 11 strategies for emotion regulation in pleasant events. The items were the same as in the daily diary and for the parent. Participants were asked on an 11-point scale ranging from 0 (never) to 10 (extremely often) how often they were performing these activities or thoughts:
   15. I think that the situation is a very special moment. (*Reappraisal as special*, hence a cognitive reappraisal strategy)
   16. I am distracted and busy myself with other things. (*Distraction*)
   17. I think about how my strengths contributed to the situation. (*Self-reinforcement*, hence a cognitive reappraisal strategy)
   18. I search for ‘the fly in the soup’. (the common German saying: “Ich suche das Haar in der Suppe”) (*Attention to negatives*)
   19. I think about the situation repeating itself or having positive consequences. (*Reappraisal as more positive*, thus a cognitive reappraisal strategy)
   20. I pay full attention to the situation. (*Directing attention*)
   21. I remember situations which were just as pleasant. (*Positive similar event*, hence a cognitive reappraisal strategy)
   22. I let myself enjoy the moment. (*Enjoying the moment*)
   23. I clearly express my pleasant emotions. (*Expression of emotions*)
   24. I actively try to make the event even more pleasant. (*Situation modification*)
   25. I tell others about the pleasant event. (*Social sharing*)
   26. An abbreviated version of the Multidimensional State Mindfulness Questionnaire, for the two subscales of nonjudgmental acceptance and present-moment attention (Blanke & Brose, 2017).
   27. A questionnaire for assessing empathy, which was an abbreviated version of the Interpersonal Reactivity Index, with the two subscales Perspective Taking and Empathic Concern (Davis, 1983; Neumann et al., 2003)
   28. The short version of the Self-Compassion Scale (Raes et al., 2011)
   29. Regarding the adolescent that participates in the study: Six questions assessing emotional warmth in parenting and four questions regarding inconsistency in parenting (adapted from Mönkediek et al., 2020).
   30. Regarding the adolescent that participates in the study: One item assessing closeness to the adolescent and two items assessing conflicts with the adolescent (self-developed by Ute Kunzmann)
   31. Regarding the adolescent who participates in the study: The autonomy scale of the Perceptions of Parents Scale (adapted from Grolnick et al., 1991), which we translated into German with the help of an English native speaker with an excellent level of German.
6. Finally, the parents were thanked for their participation and informed that they would receive 4€ as compensation for their efforts. They could choose that either their adolescent would receive this money – or that they should receive it themselves, in which case they had to provide some bank details.

## Introductory Session

1. As this is the first part of the study for the adolescent, the experimenter welcomed the participant and asked him/her to fill out an informed consent for himself/herself and, for participants younger than 18 years, to also provide informed consent from a parent with custody. Once acquired, the experimenter gave the participants more information about the objective of the study, which was to better understand how adolescents regulate their emotions, and to give them an overview of the components of the study while describing them in detail to the participants. Additionally, participants were given a small booklet with detailed information about the study. Finally, participants were – as already done during the call when they were first recruited – asked if they truly had no irregular events occurring during the time of the daily diary (such as vacations or surgery), and then appointments were made (for example, for the debriefing session).
2. Next, participants were told that they would undergo a number of ‘quiz tasks’ that they should try their best in. These were the following cognitive performance tests:

a) One test of perceptional speed, in which participants had to draw as many symbols as quickly as possible according to a certain digit-to-symbol scheme (Lindenberger et al., 1993).

b) One test of word fluency speed, in which participants should name as many animals as possible within 90 seconds (Lindenberger et al., 1993).

c) One test of auditory working memory, of the Wechsler Intelligence Scale – 5^th^ edition (Petermann, 2017), in which participants had to remember digits and repeat them in a forward, backward, and ascending manner. As none of the 125 participants managed to complete the tasks of the hardest difficulty, no ceiling effect emerged.

d) One test of verbal understanding and conceptual knowledge, also of the Wechsler Intelligence Scale – 5^th^ edition (Petermann, 2017), in which participants had to correctly explain certain words or concepts (such as ‘What role is the human heart fulfilling in the human body?’).

1. In the following, participants were told that they should fill out some questionnaires on a laptop provided by the experimenter. They were asked if the experimenter could sit next to them to be able to help them in case any questions arose. Next, participants answered a few questions regarding their sociodemographic status, thus about their age, gender (female, male, other, I don’t want to respond), whether they currently attend school, which type of school they were visiting or had visited last, their current main activity (such as doing a traineeship, attending school, studying at university, working), if they were in a relationship, who they were living together with and the number of siblings. Then they were asked about their life satisfaction. At this point, the experimenter asked them if they had any more questions and told them that there were no such answers as ‘incorrect’ or ‘correct’ ones and that participants should, after they have read the instructions, answer intuitively. Then, to ensure the participants’ privacy, the experimenters told them to fill out the following questionnaires on the laptop on their own, while the experimenter would be in an adjacent room. In case some questions or answer options turned out to be unclear, the participant should go to the experimenter and ask. Otherwise, the participant should approach the experimenter once finished. The questionnaires the participants filled out were the following:
2. A single item assessing life satisfaction (SOEP Group, 2023). This was the same as for the parent.
3. An abbreviated version of the Positive and Negative Affect Schedule (Krohne et al., 1996; Watson & Clark, 1988), 17 items, eight additional items assessing positive and negative affect, as well as the five items for assessing depressive symptoms. This was the same as for the parent.
4. An abbreviated version of the Multidimensional State Mindfulness Questionnaire, for the two subscales of nonjudgmental acceptance and present-moment attention (Blanke & Brose, 2017). This was the same as for the parent.
5. A newly created inventory of 10 strategies for emotion regulation in unpleasant events. These were the same as in the daily diary. Participants were asked on an 11-point scale ranging from 0 (never) to 10 (extremely often) how often they were performing certain activities or thoughts. As these were the same as for the parent, they shall not be repeated here.
6. A newly created inventory of 11 strategies for emotion regulation in pleasant events. These were the same as in the daily diary. This was the same as for the parent. Participants were asked on an 11-point scale ranging from 0 (never) to 10 (extremely often) how often they were performing certain activities or thoughts. As these were the same as for the parent, they shall not be repeated here.
7. A questionnaire for assessing empathy, which was an abbreviated version of the Interpersonal Reactivity Index, with the two subscales Perspective Taking and Empathic Concern (Davis, 1983; Neumann et al., 2003).
8. The Prosociality Scale (Caprara et al, 2005; Fassbender & Luhmann, 2021).
9. The short version of the Self Compassion Scale (Raes et al., 2011).
10. A questionnaire for assessing depressive symptoms, the Patient Health Questionnaire-9 – which consisted of eight items here, as we omitted the question about suicidal ideation out of ethical concerns (Gräfe et al., 2004; Kocalevent et al., 2013; Spitzer et al., 1999).
11. Three questions from validated clinical interviews (Margraf et al., 2017a; 2017b; Schneider et al., 2017) about 1) whether the participant received professional diagnostics for a psychological problem or a mental disorder in the last six months, 2) whether the participant is currently in professional treatment, such as psychotherapy, for a mental disorder, 3) whether the participant has ever received ambulatory or stationary treatment for a mental disorder. The second question was used to discern between participants with and without a mental disorder.
12. A single question, in which participants could specify any number of parent(s) they are in regular contact with. The options were mother, stepmother, father, stepfather, no more contact anymore, or other (which could be specified later on, for example for adoptive parents).
13. A single question inquiring which parent filled out the parents’ questionnaires. Depending on the participants’s answer, they received the following questionnaires for the person they indicated – with the questions tailored to the respective person (for example: ‘How often does your mother do the following?’/’How often does your stepfather do the following?’).
14. Six questions assessing emotional warmth in parenting and four questions regarding inconsistency in parenting (Mönkediek et al., 2020).
15. One item assessing closeness to the parent and two items assessing conflicts with the parent (self-developed by Ute Kunzmann).
16. The autonomy scale of the Perceptions of Parents Scale (Grolnick et al., 1991), which we translated into German, with the help of an English native speaker with an excellent level of German.
17. Finally, participants were introduced to the daily diary. They were given information about when to fill it out (after 8 pm each evening and before going to bed, in a quiet moment without anybody around), that they would receive an e-mail each evening with the link in it, and that in case of technical problems or any questions, they could call the experimenter on their mobile phone, even during nighttime. After they had been asked which device they would use most often to fill out the daily diary (smartphone/tablet/laptop), they would sit down with the experimenter in front of their preferred device while guiding them through an entry of the daily diary as if it was their first entry (which it was not, since this data was not evaluated). On each page of the daily diary, the experimenter explained in detail the question(s) and occasionally also the answer formats. The greatest care was put into instructions about the emotion regulation strategies to ensure that participants had correctly understood each strategy. Once completed, the participants were asked once again if they had any more questions regarding the daily diary and then were finally released, with their first entry of the daily diary starting one to seven days ahead.

## Daily Diary

1. The participants filled out the daily diary on their own for 28 consecutive days. In rare instances, in which participants had an irregular event happening during these 28 days which could not be rescheduled (for example, a music camp during the weekend), they were instructed to not fill out the diary during these days – instead, the daily diary would be extended by the number of days missed. Technical problems occurred very rarely – most of these could be resolved by the experimenter team, while those few that could not be resolved could be attributed to problems with the servers of the platform which hosted the webpage of the daily diary, formr 0.19.4 and 0.20.6 (Arslan et al., 2020).
2. In the following, we will list all the items of the daily diary and the respective answer formats. To avoid participants accidentally answering incorrectly, we kept the order of items unchanged throughout the 28 days of the daily diary. Please note: Forced choice answers are coded as (1), (2),…, (99). Multiple choice answers are marked by letters (a), (b),…, (z).

| **Item Description** | **Response Format** |
| --- | --- |
| **Welcome to the [survey number]th daily survey!** | Instruction |
| Please enter your initials and the year of your birth (e.g. for Anna Baecker, born 2007, the code would be AB2007). | Open response format |
| **Your feelings at the current moment**  The first part is about your feelings right now after the day you have experienced today. For each of the following feelings, indicate how intensely you are experiencing it at the current moment.  If you are not experiencing a feeling at all, please enter ‘0’. | Instruction |
| satisfied | 0 (not at all) – 10 (extremely intensely) |
| sad | 0 (not at all) – 10 (extremely intensely) |
| afraid | 0 (not at all) – 10 (extremely intensely) |
| upset | 0 (not at all) – 10 (extremely intensely) |
| optimistic | 0 (not at all) – 10 (extremely intensely) |
| guilty | 0 (not at all) – 10 (extremely intensely) |
| worried | 0 (not at all) – 10 (extremely intensely) |
| joyful | 0 (not at all) – 10 (extremely intensely) |
| depressed | 0 (not at all) – 10 (extremely intensely) |
| interested | 0 (not at all) – 10 (extremely intensely) |
| attentive | 0 (not at all) – 10 (extremely intensely) |
| irritable | 0 (not at all) – 10 (extremely intensely) |
| full of energy | 0 (not at all) – 10 (extremely intensely) |
| at the current moment: Satisfied with my life | 0 (not at all) – 10 (extremely intensely) |
| at the current moment: Satisfied with my day | 0 (not at all) – 10 (extremely intensely) |
| **Unpleasant event of the day**  Please think of a situation that was unpleasant for you today. If you can't think of a situation at first, please remember that situations that only lasted a short time and were not very intense also count (for example a thought, a missed bus, a meal that didn't taste good, an unkind gesture). Most people experience a large number of such situations or events during the day. In this study, we are interested in precisely these situations.  If there were several such situations, please choose the situation that was the most unpleasant for you.  Please tap the ‘Next’ button. | Instruction |
| Please give a brief note of what the situation was about. A short sentence or a keyword will suffice.  Please enter your answer in the box below this text. You are welcome to make a short note that only you understand, but please enter something in this field. | Open response format (5 letters minimum) |
| Please select how the unpleasant event can be categorised the best.  Please select only one option. | Own health (1)  Finances/money (2)  Worries about others (3)  Tensions or conflicts with others (4)  Missing/having lost someone or something (5)  Pressure to perform (6)  Being over-challenged/Time pressure (7)  Adversities of everyday life (e.g. mishaps, delays) (8)  Society/politics (9)  Other (10) 🡪 If chosen, it had to be specified next |
| What was the situation mostly about?  Please select only one option. | It was about being unsettled or threatened by someone or something (1)  It was about a loss or something I can not have or can not experience (2)  It was about something that I mistakenly did or mistakenly did not do (3)  It was about something that others mistakenly did or mistakenly did not do (4)  Other (5) 🡪 If chosen, it had to be specified next |
| **Activity during the situation**  Please select what your main activity in the situation has been.  Please select only one option. | Activity in the house or garden (1)  On the way to a place (2)  Out-of-home errands (e.g. shopping, bank, hairdresser, doctor) (3)  Socialising (4)  Sporting activity (5)  Leisure activity, hobby (6)  Cultural event (e.g. visiting a concert, a museum, a cinema) (7)  Occupation, training, school or voluntary work (8)  Media consumption (e.g. social media, TV) (9)  Recreation (10)  Other (11) 🡪 If chosen, it had to be specified next |
| Please indicate at approximately what time the situation took place today.  For this, please first enter the full hour using the menu. To open the menu, please tap on the small clock. Then enter the minutes by hand. | Filling in the time (format hh:mm) |
| Please enter the approximate duration of the situation.  For this, please enter the duration of the situation in minutes in the field below this text. | Whole number bigger than 0 |
| Please indicate who else was involved in or played a role in the situation apart from you.  Multiple options may be selected. | Nobody but me (a)  Partner (b)  Members of the immediate family (c)  Relatives (d)  Friends (e)  More distant acquaintances (f)  Classmates, colleagues (g)  Superiors, teachers (h)  Employees, my patients/clients/customers/pupils (i)  Persons providing services (e.g. doctors, hairdressers) (j)  Strangers (k)  Other (l) 🡪 If chosen, it had to be specified next |
| **Your feelings in the situation**  Below we present you with a series of words that describe different feelings. Read each word and then indicate what feelings the situation triggered. Please indicate how intensely you have experienced each of the following feelings in the situation.  If you did not experience a feeling at all, please enter ‘0’. | Instruction |
| sad | 0 (not at all) – 10 (extremely intensely) |
| afraid | 0 (not at all) – 10 (extremely intensely) |
| upset | 0 (not at all) – 10 (extremely intensely) |
| optimistic | 0 (not at all) – 10 (extremely intensely) |
| guilty | 0 (not at all) – 10 (extremely intensely) |
| interested | 0 (not at all) – 10 (extremely intensely) |
| worried | 0 (not at all) – 10 (extremely intensely) |
| depressed | 0 (not at all) – 10 (extremely intensely) |
| attentive | 0 (not at all) – 10 (extremely intensely) |
| dissatisfied | 0 (not at all) – 10 (extremely intensely) |
| irritable | 0 (not at all) – 10 (extremely intensely) |
| tense | 0 (not at all) – 10 (extremely intensely) |
| Please indicate what you thought or did in the situation to influence your feelings. For each of the following strategies, please indicate the extent to which you used the strategy in the situation.  If you did not use a strategy at all, please enter ‘0’. | Instruction |
| I contemplated how I could solve the problem. | 0 (not at all) – 10 (extremely intensely) |
| I thought about how my strengths could help me in this situation. | 0 (not at all) – 10 (extremely intensely) |
| I tried viewing the situation in a more positive light. | 0 (not at all) – 10 (extremely intensely) |
| I thought that the situation might not turn out that bad. | 0 (not at all) – 10 (extremely intensely) |
| I got active and tried changing the situation for the better. | 0 (not at all) – 10 (extremely intensely) |
| I distracted myself and directed my attention to other things. | 0 (not at all) – 10 (extremely intensely) |
| I did not let my unpleasant emotions show. | 0 (not at all) – 10 (extremely intensely) |
| I thought about the situation in a detached manner. | 0 (not at all) – 10 (extremely intensely) |
| I imagined how bad the situation might still get. | 0 (not at all) – 10 (extremely intensely) |
| I paid full attention to the situation. | 0 (not at all) – 10 (extremely intensely) |
| How well did you manage to influence your feelings? | 0 (not at all) – 10 (extremely well) |
| **Your assessment of the situation** | Instruction |
| How much were you responsible for the situation? | 0 (not at all) – 10 (extremely) |
| How much were others responsible for the situation? | 0 (not at all) – 10 (extremely) |
| How well did you think you could exert influence on the situation? | 0 (not at all) – 10 (extremely) |
| How well were you able to foresee the situation coming up? | 0 (not at all) – 10 (extremely) |
| How serious is the problem that arose in the situation for you? | 0 (not at all) – 10 (extremely) |
| How typical are situations like these for your current everyday life? | 0 (not at all) – 10 (extremely) |
| How important is it for you what happened in the situation? | 0 (not at all) – 10 (extremely) |
| **Pleasant event of the day**  Now let's have a look at another situation that you experienced today.  Please think of a situation that was pleasant for you today. If you can't think of a situation at first, please remember that situations that only lasted a short time and were not very intense also count (for example a friendly gesture, a delicious meal, a good conversation). Most people experience a large number of such situations or events during the day. In this study, we are interested in precisely these situations.  If there were several such situations, please choose the situation that was the most pleasant for you.  Please tap the ‘Next’ button. | Instruction |
| Please give a brief note of what the situation was about. A short sentence or a keyword will suffice.  Please enter your answer in the box below this text. You are welcome to make a short note that only you understand, but please enter something in this field. | Open response format (5 letters minimum) |
| Please select how the pleasant event can be categorised the best.  Please select only one option. | Achieved something important (1) Having been there for others, having supported them (2) Having received support and encouragement from others (3) Intimacy, closeness, enjoying another’s company (4)  Learned/experienced something new or exciting (5)  New, interesting encounter (6) Other (7) 🡪 If chosen, it had to be specified next |
| **Activity during the situation**  Please select what your main activity in the situation has been.  Please select only one option. | Activity in the house or garden (1)  On the way to a place (2)  Out-of-home errands (e.g. shopping, bank, hairdresser, doctor) (3)  Socialising (4)  Sporting activity (5)  Leisure activity, hobby (6)  Cultural event (e.g. visiting a concert, a museum, a cinema) (7)  Occupation, training, school or voluntary work (8)  Media consumption (e.g. social media, TV) (9)  Recreation (10)  Other (11) 🡪 If chosen, it had to be specified next |
| Please indicate at approximately what time the situation took place today.  For this, please first enter the full hour using the menu. To open the menu, please tap on the small clock. Then enter the minutes by hand. | Filling in the time (format hh:mm) |
| Please enter the approximate duration of the situation.  For this, please enter the duration of the situation in minutes in the field below this text. | Whole number bigger than 0 |
| Please indicate who else was involved in or played a role in the situation apart from you.  Multiple options may be selected. | Nobody but me (a)  Partner (b)  Members of the immediate family (c)  Relatives (d)  Friends (e)  More distant acquaintances (f)  Classmates, colleagues (g)  Superiors, teachers (h)  Employees, my patients/clients/customers/pupils (i)  Persons providing services (e.g. doctors, hairdressers) (j)  Strangers (k)  Other (l) 🡪 If chosen, it had to be specified next |
| **Your feelings in the situation**  Below we present you with a series of words that describe different feelings. Read each word and then indicate what feelings the situation triggered. Please indicate how intensely you have experienced each of the following feelings in the situation.  If you did not experience a feeling at all, please enter ‘0’. | Instruction |
| satisfied | 0 (not at all) – 10 (extremely intensely) |
| relaxed | 0 (not at all) – 10 (extremely intensely) |
| worried | 0 (not at all) – 10 (extremely intensely) |
| attentive | 0 (not at all) – 10 (extremely intensely) |
| guilty | 0 (not at all) – 10 (extremely intensely) |
| interested | 0 (not at all) – 10 (extremely intensely) |
| joyful | 0 (not at all) – 10 (extremely intensely) |
| irritable | 0 (not at all) – 10 (extremely intensely) |
| proud | 0 (not at all) – 10 (extremely intensely) |
| grateful | 0 (not at all) – 10 (extremely intensely) |
| depressed | 0 (not at all) – 10 (extremely intensely) |
| optimistic | 0 (not at all) – 10 (extremely intensely) |
| Please indicate what you thought or did in the situation to influence your feelings. For each of the following strategies, please indicate the extent to which you used the strategy in the situation.  If you did not use a strategy at all, please enter ‘0’. | Instruction |
| I thought that the situation is a very special moment. | 0 (not at all) – 10 (extremely intensely) |
| I was distracted and busied myself with other things. | 0 (not at all) – 10 (extremely intensely) |
| I thought about how my strengths contributed to the situation. | 0 (not at all) – 10 (extremely intensely) |
| I searched for ‘the fly in the soup’. | 0 (not at all) – 10 (extremely intensely) |
| I thought about the situation repeating itself or having positive consequences. | 0 (not at all) – 10 (extremely intensely) |
| I paid full attention to the situation. | 0 (not at all) – 10 (extremely intensely) |
| I remembered situations which were just as pleasant. | 0 (not at all) – 10 (extremely intensely) |
| I let myself enjoy the moment. | 0 (not at all) – 10 (extremely intensely) |
| I clearly expressed my pleasant emotions. | 0 (not at all) – 10 (extremely intensely) |
| I actively tried to make the event even more pleasant. | 0 (not at all) – 10 (extremely intensely) |
| I told others about the pleasant event. | 0 (not at all) – 10 (extremely intensely) |
| How well did you manage to influence your feelings? | 0 (not at all) – 10 (extremely well) |
| **Your assessment of the situation** | Instruction |
| How much were you responsible for the situation? | 0 (not at all) – 10 (extremely) |
| How much were others responsible for the situation? | 0 (not at all) – 10 (extremely) |
| How well did you think you could exert influence on the situation by yourself? | 0 (not at all) – 10 (extremely) |
| How well were you able to foresee the situation coming up? | 0 (not at all) – 10 (extremely) |
| How typical are situations like these for your current everyday life? | 0 (not at all) – 10 (extremely) |
| How important is it for you what happened in the situation? | 0 (not at all) – 10 (extremely) |
| **Your day as a whole**  Finally, we would like to ask you a few questions about your day in general. This is not about specific situations that you have experienced today but about your overall impression of the day.  Please tap the ‘Next’ button. | Instruction |
| Was your day stressful? | 0 (not at all) – 10 (extremely) |
| Was today typical of your everyday life? | 0 (not at all) – 10 (extremely) |
| Unpleasant events today | 0 (very few) – 10 (extremely many) |
| Pleasant events today | 0 (very few) – 10 (extremely many) |
| **Now once again please think back to the day as a whole.**  How did you behave, and what was going on inside of you? The following questions relate to this. | Instruction |
| I thought that I could have behaved better in certain moments. | 0 (doesn’t fit at all) – 10 (fits very well) |
| I focussed on what I was doing. | 0 (doesn’t fit at all) – 10 (fits very well) |
| I focussed my attention on the current moment. | 0 (doesn’t fit at all) – 10 (fits very well) |
| I focussed on what was happening at the time. | 0 (doesn’t fit at all) – 10 (fits very well) |
| I focussed my attention on each current moment. | 0 (doesn’t fit at all) – 10 (fits very well) |
| Things went through my mind that really shouldn't be on my mind. | 0 (doesn’t fit at all) – 10 (fits very well) |
| I tried to help other people today or make them feel better (e.g. offering advice, comfort or support). | 0 (doesn’t fit at all) – 10 (fits very well) |
| Other people have helped or contributed to making me feel better today (e.g. offered advice, comfort or support). | 0 (doesn’t fit at all) – 10 (fits very well) |
| I felt connected to other people today. | 0 (I felt very disconnected) – 10 (I felt very connected) |
| These were all the questions for today. Thank you very much! | Button for closing the diary entry |

## Debriefing

1. One to seven days after having finished the daily diary, participants were debriefed in person by an experimenter. Most of the time was the same experimenter they had had during their introductory session. At first, they filled out a brief questionnaire that assessed their level of understanding of the items in the daily diary as well as how representative their days were. Level of understanding was very good (scale of 1-5: *M* = 4.25 , *SD* = 0.72) with only 3 of the 122 participants reporting of low level of understanding. At the same time, the vast majority of participants reported that their days during the daily diary had been very representative (scale of 1-5: *M* = 4.24, *SD* = 0.70), with only 2 participants having reported a low level of representativeness.
2. Next, participants were asked to fill out some more questionnaires on a laptop provided by the experimenter. These questionnaires were the following:
3. A single item assessing satisfaction with social relationships (Huinink et al., 2011)
4. Two items assessing the perceived level of received social support, based on the Berlin Social Support Scale (Schulz & Schwarzer, 2004)
5. Three items assessing the level of loneliness, based on Hughes et al. (2004) and SOEP Group, 2023)
6. One item assessing the level of the perceived overall health (Katzorreck & Kunzmann, 2018)
7. A questionnaire for assessing dispositional optimism and pessimism, the Life-Orientation-Test, Revised (Glaesmer et al., 2008)
8. A questionnaire assessing control strategies, by using an abbreviation version the short form of the Optimization in Primary and Secondary Control Scales (Heckhausen et al., 1998)
9. The Goal Adjustment Scale (Haase & Wrosch, 2020; Wrosch et al., 2003)
10. The questionnaire on Epistemological Understanding (Kuhn et al. 2000)
11. The General Intellectual Humility Scale (Leary et al., 2017)
12. Another questionnaire assessing intellectual humitly, namely the Limitations-Owning Intellectual Humility Scale (Haggard et al., 2018)
13. Next, participants were told for how many days they had filled out the daily diary, if they would receive the 10€ bonus for having filled out the daily diary for at least 24 days, whether they would receive the additional 4€ of the questionnaires one of their parents had filled out and, accordingly, the final sum. The underlying calculation was that participants would receive 9.50€ as compensation for one hour of work, with the study taking slightly less than 9.5 hours, to which the aforementioned bonuses were added on top. Hence, participants could receive up to 104€. Participants who were students of psychology could, if they wish, instead receive 9,5 hours of study credit and 14€ as compensation. Participants would either receive the money in cash or, more commonly, have it transferred to their or their parent’s bank account, for which they had to provide the necessary banking details. Finally, participants were once again thanked for their participation and bade farewell.

# References

Arslan, R. C., Walther, M. P., & Tata, C. S. (2020). formr: A study framework allowing for automated feedback generation and complex longitudinal experience-sampling studies using R. *Behavior Research Methods*, 52, 376–387. <https://doi.org/10.3758/s13428-019-01236-y>

Glaesmer, H., Hoyer, J., Klotsche, J., & Herzberg, P. Y. (2008). Die deutsche Version des Life-Orientation-Tests (LOT-R) zum dispositionellen Optimismus und Pessimismus. *Health Psychology, 16*(1), 26–31. <https://doi.org/10.1026/0943-8149.16.1.26>

Gräfe, K., Zipfel, S., Herzog, W., & Löwe, B. (2004). Screening psychischer Störungen mit dem “Gesundheitsfragebogen für Patienten (PHQ-D)“. *Diagnostica, 50*(4), 171-181. <https://doi.org/10.1026/0012-1924.50.4.171>

Haase, C. M., & Wrosch, C. (2020). *German translation of the Goal Adjustment Scale.* <https://doi.org/10.31234/osf.io/k4zev>

Haggard, M., Rowatt, W. C., Leman, J. C., Meagher, B., Moore, C., Fergus, T., Whitcomb, D., Battalay, H., Baehr, J., & Howard-Snyder, D. (2018). Finding middle ground between intellectual arrogance and intellectual servility: Development and assessment of the limitations-owning intellectual humility scale. *Personality and Individual Differences, 124*(1), 184-193. <https://doi.org/10.1016/j.paid.2017.12.014>

Heckhausen, J., Schulz, R., & Wrosch, C. (1998). *Developmental regulation in adulthood: Optimization in primary and secondary control. A Multiscale Questionnaire.* Max Planck Institute for Human Development.

Huinink, J., Brüderl, J., Nauck, B., Walper, S., Castiglioni, L., & Feldhaus, M. (2011). Panel Analysis of Intimate Relationships and Family Dynamics (pairfam): Conceptual framework and design. *Journal of Family Research, 23*(1), 77–101. <https://doi.org/10.20377/jfr-235>

Katzorreck, M. & Kunzmann, U. (2018). Greater empathic accuracy and emotional reactivity in old age: The sample case of death and dying. *Psychology and Aging, 33*(8), 1202–1214. <https://doi.org/10.1037/pag0000313>

Kocalevent, R. D., Hinz, A., & Brähler, E. (2013). Standardization of the depression screener patient health questionnaire (PHQ-9) in the general population. *General hospital psychiatry*, *35*(5), 551-555. <https://doi.org/10.1016/j.genhosppsych.2013.04.006>

Krohne, H. W., Egloff, B., Kohlmann, C.-W., & Tausch, A. (1996). Untersuchung mit einer deutschen Form der Positive and Negative Affect Schedule (PANAS). *Diagnostica, 42*(2), 139-156. <https://doi.org/10.1037/t49650-000>

Kuhn, D., Cheney, R., & Weinstock, M. (2000). The development of epistemological understanding. *Cognitive development, 15*(3), 309-328. <https://doi.org/10.1016/S0885-2014(00)00030-7>

Leary, M. R., Diebels, K. J., Davisson, E. K., Jongman-Sereno, K. P., Isherwood, J. C., Raimi, K. T., Deffler, S. A., & Hoyle, R. H. (2017). Cognitive and interpersonal features of intellectual humility. *Personality and Social Psychology Bulletin, 43*(6), 793-813. <https://doi.org/10.1177/0146167217697695>

Margraf, Cwik, Pflug &Schneider (2017a). Structured clinical interviews for mental disorders across the lifespan: Psychometric quality and further developments of the DIPS Open Access interviews. [Strukturierte klinische Interviews zur Erfassung psychischer Störungen über die Lebensspanne: Gütekriterien und Weiterentwicklungen der DIPS-Verfahren.] *Zeitschrift für Klinische Psychologie und Psychotherapie, 46*(3).

Margraf, J., Cwik, J. C., Suppiger, A. & Schneider, S. (2017b). *DIPS Open Access: Diagnostic Interview for Mental Disorders. [DIPS Open Access: Diagnostisches Interview bei psychischen Störungen.]* Bochum: Mental Health Research and Treament Center, Ruhr-Universität Bochum. <https://doi.org/10.13154/rub.100.89>

Mönkediek, B., Schulz, W., Eichhorn, H., & Diewald, M. (2020). Is there something special about twin families? A comparison of parenting styles in twin and non-twin families. *Social Science Research, 90*, 102441.

Petermann, F. (Ed.). (2017). *Wechsler Intelligence Scale for Children – Fifth Edition (WISC-V). Deutsche Übersetzung und Adaption des WISC-V von D. Wechsler*. Pearson.

Schneider, S., Pflug, V., In-Albon, T. & Margraf, J. (2017). *Kinder-DIPS Open Access: Diagnostisches Interview bei psychischen Störungen im Kindes- und Jugendalter.* Bochum: Forschungs- und Behandlungszentrum für psychische Gesundheit, Ruhr-Universität Bochum. <https://doi.org/10.13154/rub.100.89>

Schulz, U. & Schwarzer; R. (2004). Long-term effects of spousal support on coping with cancer after surgery. *Journal of Social and Clinical Psychology, 23*(5), 716–732. <https://doi.org/10.1521/jscp.23.5.716.50746>

Spitzer, R. L., Kroenke, K., Williams, J. B. & The Patient Health Questionnaire Primary Care Study Group (1999). Validation and utility of a self-report version of PRIME-MD: The PHQ primary care study. *JAMA, 282*(18), 1737–1744. <https://doi.org/10.1001/jama.282.18.1737>

SOEP Group (2023). SOEP-Core – 2021: Person (M3-M6, Wiederbefragte, mit Verweis auf Variablen). *SOEP Survey Papers 1278: Series A – Survey Instruments (Erhebungsinstrumente).* Berlin: DIW Berlin/SOEP. <https://doi.org/10419/280377>

Wrosch, C., Scheier, M. F., Miller, G. E., Schulz, R., & Carver, C. S. (2003). Adaptive self-regulation of unattainable goals: Goal disengagement, goal reengagement, and subjective well-being. *Personality and Social Psychology Bulletin, 29*(12), 1494-1508. <https://doi.org/10.1177/0146167203256921>
